# Depth distribution of plant-parasitic nematodes on bentgrass golf greens in Missouri and Indiana

**DOI:** 10.2478/jofnem-2024-0006

**Published:** 2024-03-20

**Authors:** Asa L. McCurdy, Jefferson Barizon, G.L. Miller

**Affiliations:** Purdue University, Dept. of Botany and Plant Pathology, 915 Mitch Daniels Blvd, West Lafayette, IN 47907; University of Missouri, SCN Diagnostics Clinic, 1054 E. Campus Loop, Columbia, MO 65211

**Keywords:** *Agrostis stolonifera*, *Criconemoides* spp., *Hoplolaimus galeatus*, *Hoplolaimus magnistylus*, *Hoplolaimus* spp., *Hoplolaimus stephanus*, lance nematode, management, *Meloidogyne graminicola*, *Meloidogyne marylandi*, *Meloidogyne naasi*, *Meloidogyne* spp., molecular biology, nematicide, population dynamics, root-knot nematode, ring nematode, turfgrass, vertical distribution

## Abstract

Control of plant-parasitic nematodes (PPNs) on golf putting greens with nematicides is dependent on the seasonal occurrence and depth distribution of target PPN populations. This study aimed to determine if plant-parasitic nematode populations on golf course putting greens in Missouri and Indiana peaked at a targetable depth at a specific time in the year, focusing primarily on lance (*Hoplolaimus* spp.) and root-knot (*Meloidogyne* spp.) nematodes. To elucidate species diversity in the region, rDNA from a subset of lance and root-knot nematodes was sequenced and analyzed, with additional micromorphology of a lance nematode assessed in scanning electron micrographs (SEM). Soil samples were taken to a depth of 25 cm and stratified into 5 cm increments during April, June, August and October at seven sites across Missouri, three in the Kansas City metro of Kansas in 2021 and in ten sites across Indiana in 2022. Samples were stratified in five-centimeter increments and aggregated for a total of 100 cm^3^ of soil at each depth for each sampling. Samples were processed using a semi-automatic elutriator followed by the sucrose-flotation method, and populations were counted using a hemocytometer and recorded. For molecular characterization, rDNA was extracted and analyzed from 31 individual lance nematodes from one site in Missouri and eight sites in Indiana, and 13 root-knot nematodes from nine sites across Indiana. A significant interaction occurred between sampling month and depth for lance and ring nematodes Missouri/KS, with both PPN populations peaking at the 0–5 cm depth during October, which is well after most targeted nematicide applications are applied. Ring nematodes in Indiana did not follow this trend and were most abundant in August at a depth of 0–5 cm. No significant interaction between depth and month occurred for lance or root-knot nematodes in Indiana, or root-knot nematodes in Missouri/KS. *Hoplolaimus stephanus* and *H. magnistylus* were the lance species identified on golf greens, and *Meloidogyne naasi*, *M. graminicola* and *M. marylandi* were the root-knot species identified. Scanning-electron micrographs confirmed morphological characteristics unique to *H. stephanus*.

Plant-parasitic nematodes (PPNs) pose a significant risk to the health and visual quality of high amenity turfgrasses, particularly golf course putting greens which are constructed with a conducive sand-based root zone and are under intense stress from low mowing and aggressive maintenance practices (4). Control of PPNs on golf greens typically relies on the broadcast application of a nematicide. Many of these nematicide active ingredients, such as abamectin, have a low water solubility (K_s_ abamectin = 7.8 μg L^−1^) and high soil-organic carbon partitioning coefficient (K_OC_ abamectin = 4000 mL g^−1^), leading to the molecule being tied up in the organic-matter rich thatch layer of golf course putting greens (11,31). As a result of this limited mobility, contact and subsequent control of a target PPN residing deeper in the soil profile is unlikely. As a further complication, the various species of PPNs specifically parasitic to turfgrass can be present both outside and inside the root, existing as migratory ecto-, sedentary endo- or semi-endoparasites. To combat limitations in nematicide efficacy, determining if and when PPN populations aggregate to a targetable depth is paramount.

Root-knot nematodes (*Meloidogyne* Göldi, 1887) are the most economically damaging PPN of horticultural crops due to their particularly explosive and destructive endoparasitic reproductive process and ability to quickly produce new progeny (36). The explosive nature of root-knot reproduction results in epidemics that can lead to rapid decline of golf putting greens if proper control methods are not implemented in a timely manner. Morphology of root-knot nematodes is dynamic through their life cycle with gender specific characteristics developing after 3–8 weeks of plant-parasitism (7). However, root-knot nematodes are typically easily recognized due to the large size of females and host specificity between species (7).

*Hoplolaimus* von Daday, 1905, or the lance nematode, represents a genus of economically important PPNs to many cropping systems including corn (*Zea mays* L.), soybean (*Glycine max* L.) and a variety of turfgrass species (8,15,24). *Hoplolaimus galeatus* is cited as the major species of lance nematode that parasitizes turfgrass species in the United States (12,15,26). Alternatively, *H. stephanus* has seldom been reported parasitizing bentgrass outside the East Coast, Georgia, and on two golf course greens in the Midwestern United States (16,17). Although *H. galeatus* is a well-documented PPN on turfgrasses in the Southern region, similarities in morphological characteristics (14,27,32) and host range (19) of *Hoplolaimus* spp. may have resulted in over-representation of this species.

As a migratory ecto- and semi-endoparasitic PPN, the lance nematode feeds on both external and internal root tissue throughout its life cycle (29). Populations of lance nematodes on golf course putting greens have been observed to be either aggregated to the upper 5 cm of soil or evenly distributed throughout the upper 10 cm (34). Turfgrass parasitic root-knot nematode species, and the temperature requirements for their reproduction, have been documented; however, their vertical distribution within the soil beneath the turfgrass was not (21,13). Population shifts in agronomic crops such as peanuts and cotton have been recorded (25), while studies aimed at determining population fluctuation trends in turfgrass, particularly on bentgrass putting greens in the Midwest region, have yet to be conducted.

Effectively timing management strategies, particularly nematicide applications, require knowledge of the biology and seasonal occurrence of PPN populations. Nematicide applications timed when PPNs are shallow in the soil profile and just prior to a population spike would provide the highest chance of the nematicide molecule contacting the target PPN (9). This study serves as a benchmark for determining if the vertical distribution of PPNs on golf putting greens aggregates to a targetable depth during a given month in Missouri and Indiana, while building upon the knowledge base of studies conducted in other geographic locations. Additionally, this study aimed to speciate lance and root-knot nematode species on golf courses, putting greens in the Midwestern United States, specifically Missouri and Indiana, through DNA sequencing and scanning electron microscopy.

## Materials and Methods

*Sample collection:* Twenty golf putting greens established with creeping bentgrass or a mix of creeping bentgrass and *Poa annua* were sampled in April, June, August and October of 2021 and 2022. Based on their broad geographic distribution and previously observed high lance nematode populations, seven putting green sites in Missouri and three in the Kansas City area of Kansas [KC: Mission Hills (2) and Olathe (1)] were sampled in 2021. In 2022, ten additional sites in Indiana without historic nematode population data were sampled based on their broad geographic distribution across the state (Table 1). The length of each of the putting greens was measured and four equal transects were created across the green in a “W” shaped pattern. Each transect was randomly divided by four to create three points of even distribution across the green, for a total of 12 sampling points per green. Soil samples were extracted using a 1.9 cm diameter soil probe to a depth of 25 cm. Samples were separated into five groups by depth: 0–5, 5.1–10, 10.1–15 15.1–20 and 20.1–25 cm. Each group of twelve subsamples was aggregated into a single sample (100 cm^3^) and stored in a small plastic bag at a cool temperature (<20°C) prior to analysis. Samples were shipped immediately or driven to the University of Missouri SCN Diagnostics Clinic for processing.

**Table 1:** Sampling sites from both Missouri and eastern Kansas in 2021 and Indiana in 2022

**State**	**City**	**Cultivar**	**Nematicides Previously Applied**	**Construction**	**pH**	**OM %**	**K (ppm)**	**Mg (ppm)**	**Ca (ppm)**
**MO/KC 2021**									

1	St. Louis	A4	None	USGA	6.9	1.3	40	82	471
2	St. Louis	T1/A4	None	USGA	N.A.	N.A.	N.A.	N.A.	N.A.
3	St. Louis	A1/A4	Heat-killed *Burkholderia* spp., Fluopyram	Native	N.A.	N.A.	N.A.	N.A.	N.A.
4	Columbia	A1	None	USGA	N.A.	N.A.	N.A.	N.A.	N.A.
5	Columbia	SR 1020	Heat-killed *Burkholderia* spp., Abamectin	USGA	7.8	0.6	49	126	634
6	Branson	Penn A1/A4	None	USGA	N.A.	N.A.	N.A.	N.A.	N.A.
7	Cape Girardeau	Crenshaw	Fluopyram	USGA	N.A.	N.A.	N.A.	N.A.	N.A.
8	Mission Hills	A1/A4	Fluopyram	USGA	7.3	1.4	30	72	628
9	Mission Hills	A1/A4 ~10% POA	Heat-killed *Burkholderia* spp., Abamectin	USGA	7.1	1.2	64	65	597
10	Olathe	A4/Pure Distinction	Abamectin, Fluopyram	USGA	7.2	1.3	53	62	678

**Indiana 2022**									

1	Newburgh	Cohansey	None	USGA	7.4	1.1	20	51	476
2	French Lick	Penn A1/A4	None	USGA	7.9	1.0	16	35	1335
3	Columbus	Penncross	Abamectin	Native	7.5	1.6	59	93	1373
4	Indianapolis	Penncross ~20% Poa	None	USGA	7.5	2.5	49	113	1478
5	Noblesville	A1	None	USGA	7.8	N.A.	N.A.	N.A.	N.A.
6	Chesterton	Penncross/Poa (50/50)	None	USGA	7.8	1.2	21	77	890
7	Bristol	A1/A4/Poa	Fluopyram	Native	7.8	2.2	108	105	1809
8	Fort Wayne	Penncross/Poa	None	USGA	7.6	2.6	56	84	1609
9	Fort Wayne	007	None	USGA	7.5	1.6	40	138	1546
10	West Lafayette	L 39	None	USGA	7.9	1.7	30	80	1518

*Sample processing:* All samples were homogenized before extraction. Samples from the 0–5 cm group were blended using a food processor to break up verdure, thatch, and organic material, and samples from the remaining depth groups were mixed thoroughly, breaking any soil clumps with the aid of a rolling pin. For each sample, 100 cm^3^ of soil was separated by water displacement in a 1000 ml plastic beaker filled with 400 ml of tap water and mixed before being processed using a semiautomatic elutriator (3), followed by isolation using the centrifuge flotation method. Individual PPNs were identified to the genus in a 1 ml aliquot and counted with a hemocytometer under an inverted microscope at 40x to 400x magnification. Individual PPNs were identified to genus based on general morphological characteristics such as cephalic framework, stylet, esophagus type, sexual structure and position, tail shape, and cuticle, among others, with the aid of a pictorial key to genera (20).

*Statistical analysis:* Populations at the five sampling depths were compared to assess the vertical population distribution and change in population density among months. Differences in sampling depth-group populations during different sampling months were evaluated using PROC GLIMMIX in SAS 9.4 (SAS Institute). MO/KC nematode populations were analyzed together and grouped separately from Indiana. Least squared means of PPN population per 100 cm^3^ of soil were subjected to analysis of variance in PROC GLIMMIX (SAS 9.4; SAS Institute), and means were separated using Fisher's protected LSD (*P* < 0.05). Variables included were sampling depth, sampling month and sampling depth by month, with site set as a random variable.

*Phylogenetic analysis:* A subset of lance and root-knot nematodes were selected for molecular analysis from one site in STL and ten sites in Indiana, respectively (Table 2). Thirty-two individual lance and 13 root-knot nematodes (J2) were hand-picked and placed into centrifuge tubes with 200 μL of nuclease-free water. DNA was extracted from individual lance and root-knot nematodes using the EXTRACT-N-AMP kit (Sigma, St. Louis, MO), with the manufacturer's protocol modified as seen in Ma et al. (19).

**Table 2:** Species and isolates of lance nematodes sequenced in the present study.

**DNA ID**	**Location**	**Host**	**Cultivar**	**Species**	**GenBank Accession Number**
STL.1	Kirkwood, MO	Bentgrass	A1/A4	*H. stephanus*	OR948481
STL.2	Kirkwood, MO	Bentgrass	A1/A4	*H. magnistylus*	OR948484
STL.3	Kirkwood, MO	Bentgrass	A1/A4	*H. stephanus*	OR948486
STL.4	Kirkwood, MO	Bentgrass	A1/A4	*H. stephanus*	OR948495
STL.5	Kirkwood, MO	Bentgrass	A1/A4	*H. stephanus*	OR948498
STL.6	Kirkwood, MO	Bentgrass	A1/A4	*H. magnistylus*	OR948501
1.1	Newburgh, IN	Bentgrass	Cohansey	*H. magnistylus*	OR948481
1.2	Newburgh, IN	Bentgrass	Cohansey	*H. magnistylus*	OR948496
2.1	French Lick, IN	Bentgrass	Penn A1/A4	*H. stephanus*	OR948472
2.2	French Lick, IN	Bentgrass	Penn A1/A4	*H. stephanus*	OR948480
2.3	French Lick, IN	Bentgrass	Penn A1/A4	*H. stephanus*	OR948482
2.4	French Lick, IN	Bentgrass	Penn A1/A4	*H. stephanus*	OR948500
3.1	Columbus, IN	Bentgrass	Penncross	*H. stephanus*	OR948478
3.2	Columbus, IN	Bentgrass	Penncross	*H. stephanus*	OR948493
3.3	Columbus, IN	Bentgrass	Penncross	*H. stephanus*	OR948492
4.1	Indianapolis, IN	Bentgrass/~20% Poa	Penncross	*H. stephanus*	OR948475
4.2	Indianapolis, IN	Bentgrass/~20% Poa	Penncross	*H. stephanus*	OR948485
5.1	Noblesville, IN	Bentgrass	A1	*H. stephanus*	OR948476
6.1	Chesterton, IN	Bentgrass/Poa 50/50	Penncross	*H. stephanus*	OR948477
6.2	Chesterton, IN	Bentgrass/Poa 50/50	Penncross	*H. stephanus*	OR948487
7.1	Bristol, IN	Bentgrass/~20% Poa	A1/A4	*H. stephanus*	OR948497
10.1	West Lafayette, IN	Bentgrass	L 93	*H. stephanus*	OR948473
10.2	West Lafayette, IN	Bentgrass	L 93	*H. stephanus*	OR948474
10.3	West Lafayette, IN	Bentgrass	L 93	*H. stephanus*	OR948479
10.4	West Lafayette, IN	Bentgrass	L 93	*H. stephanus*	OR948483
10.5	West Lafayette, IN	Bentgrass	L 93	*H. stephanus*	OR948489
10.6	West Lafayette, IN	Bentgrass	L 93	*H. stephanus*	OR948490
10.7	West Lafayette, IN	Bentgrass	L 93	*H. stephanus*	OR948491
10.8	West Lafayette, IN	Bentgrass	L 93	*H. stephanus*	OR948494
10.9	West Lafayette, IN	Bentgrass	L 93	*H. stephanus*	OR948499
10.10	West Lafayette, IN	Bentgrass	L 93	*H. magnistylus*	OR948502

**Root-Knot**					

**DNA ID**	**Location**	**Host**	**Cultivar**	**Species**	**GenBank Accession Number**

1.1	Newburgh, IN	Bentgrass	Cohansey	*M. gramincola*	PP034063
2.1	French Lick, IN	Bentgrass	Penn A1/A4	*M. gramincola*	PP034062
4.1	Indianapolis, IN	Bentgrass/~20% Poa	Penncross	*M. marylandi*	PP034064
4.2	Indianapolis, IN	Bentgrass/~20% Poa	Penncross	*M. naasi*	PP034066
5.1	Noblesville, IN	Bentgrass	A1	*M. graminicola*	PP034061
6.1	Chesterton, IN	Bentgrass/Poa 50/50	Penncross	*M. marylandi*	PP034071
7.1	Bristol, IN	Bentgrass/~20% Poa	A1/A4	*M. marylandi*	PP034065
7.2	Bristol, IN	Bentgrass/~20% Poa	A1/A4	*M. naasi*	PP034072
8.1	Fort Wayne, IN	Bentgrass/Poa 50/50	Penncross	*M. naasi*	PP034067
9.1	Fort Wayne, IN	Bentgrass	007	*M. naasi*	PP034069
10.1	West Lafayette, IN	Bentgrass	L 93	*M. graminicola*	PP034060
10.2	West Lafayette, IN	Bentgrass	L 93	*M. naasi*	PP034068
10.3	West Lafayette, IN	Bentgrass	L 93	*M. naasi*	PP034070

The rDNA ITS region from morphologically identified lance nematodes was amplified with genus-level primer sequences Hoc-1f (5′- AACCTGCTGCTGGATCATTA-3′) and LSUD-03r (5′- TATGCTTAAGTTCAGCGGGT-3′) and were subsequently sequenced (1). The D2/D3 region of the 28S gene from morphologically identified root-knot nematodes was amplified with primer sequences ((RK28SF (5′- CGGATAGAGTCGGCGTATC-3′) and MR (5′- AACCGCTTCGGACTTCCACCAG-3′)) designed by Ye et al. (36). Amplification was conducted in a 25 μl mixture containing 10 μl of Taq Ready Mix (Sigma), 13 μl of nuclease-free water, 1 μl of DNA and 0.5 μl of each primer. PCR amplification of the ITS rDNA region of lance nematode samples was conducted with the following cycle design: initial denaturation at 95°C for 3 min, followed by 35 cycles of 95°C for 45 s, 59°C for 1 min 30 s, 72°C for 2 min and a final extension at 72°C for 10 min. PCR amplification of the D2/D3 region of root-knot nematode samples was conducted with the following cycle design: initial denaturation at 95°C for 5 min, followed by 40 cycles of denaturation at 94° for 30 s, 55°C for 45 s, 72°C for 1 min and a final extension at 72° for 10 min. Amplification was confirmed via electrophoresis on a 1% agarose gel. Amplicons were purified with ExoSAP-IT (ThermoFisher, Waltham, MA) following the manufacturer's recommendations and sent to Eurofins Genomics (Eurofins Genomics, Louisville, KY) for sequencing. Lance and root-knot sample DNA was also amplified using species-specific primers (Table 3) developed for *H. stephanus*, *H. columbus* and *H. galeatus* (19,1) and *M. naasi*, *M. marylandi*, *M. graminis* and *M. incognita* (36), respectively. Species-specific amplicons were visualized after electrophoresis on a 1% agarose gel for further evidence of speciation.

**Table 3:** *Hoplolaimus* spp. reverse primers paired with Hoc-1f (1,19). *Meloidogyne* spp. primer pairs used in this study (36). All T_m_ (°C) fell between 55–65.

***Hoplolaimus* spp.**				

**Species**	**Primer Code**	**Primer Sequence (5′-3′)**	**T_m_ (°C)**	**Size of PCR fragment (bp)**
*Hoplolaimus spp.*	LSUD-03r	TATGCTTAAGTTCAGCGGGT	60	1,030
*H. stephanus*	Hs-1r	GCCAGTGTGTTCCGCTCGCA	63.2	260
*H. stephanus*	Hs-1f	CCTGCCTTGGGGGTCGCTTG	63.7	260
*H. columbus*	HC-1r	TCAGCACACAATGGTACCTTT	62	580
*H. galeatus*	HG-2r	TCCTCGTTCACACATTGACA	62	120

***Meloidogyne* spp.**					

*Meloidogyne spp.(F)*	RK28SF	CGGATAGAGTCGGCGTATC	55–60	612
*Meloidogyne spp.(R)*	MR	AACCGCTTCGGACTTCCACCAG			
*M. graminis(F)*	Mg28SFs	GATGTGCAGATATTTTCCGTCAAGG	55–60	198
M. graminis(R)*	RK28SUR	CCCTATACCCAAGTCAGACGAT			
*M. marylandi(F)*	MgmITSFs	GATCGTAAGACTTAATGAGCC	55–60	323
M. marylandi(R)*	RK28SUR	CCCTATACCCAAGTCAGACGAT			
*M. naasi(F)*	Mn28SFs	GTCTGATGTGCGACCTTTCACTAT	55–60	272
M. naasi(R)*	RK28SUR	CCCTATACCCAAGTCAGACGAT			
*M. incognita(F)*	Inc-K14-F	CCCGCTACACCCTCAACTTC	55–60	399
*M. incognita(R)*	Inc-K14-R	GGGATGTGTAAATGCTCCTG		

Phylogenetic trees were developed with consensus sequences using ClustalW in MEGA (Pennsylvania State University, State College, PA). The ITS sequences of *Hoplolaimus* spp. described in Holguin (16) were downloaded from GenBank and included in the phylogenetic analysis of lance nematodes. The 16S (21) and 28S (21, Tian, 2017, Ye, 2016) sequences of *Meloidogyne* spp. were downloaded from GenBank and included in phylogenetic analysis of root-knot nematodes. Both phylogenetic trees were constructed with the neighbor-joining algorithm using the Kimura two-parameter model with *Litylenchus* spp. (LC383724) as the outgroup. Bootstrap values on branch nodes are based on 1,000 random samples of the data set. *Morphological characterization*: One female lance nematode from site 5 (Table 2) was used for morphological analysis at the Purdue Electron Microscopy Facility (West Lafayette, IN). Micrographs were acquired using a FEI Nova NanoSEM (Thermo Fischer, Waltham, MA) scanning electron microscope (SEM). The lance nematode was processed with a 2.5% glutaraldehyde fixation for 15 minutes, 1% osmium tetroxide for 15 minutes, a five-minute water rinse, then a series of five-minute ethanol dehydrations (50%, 75%, 95%, 100%) followed by dehydration via hexamethyldisilazane (HMDS) and a final platinum coating.

## Results

**Figure 1:**
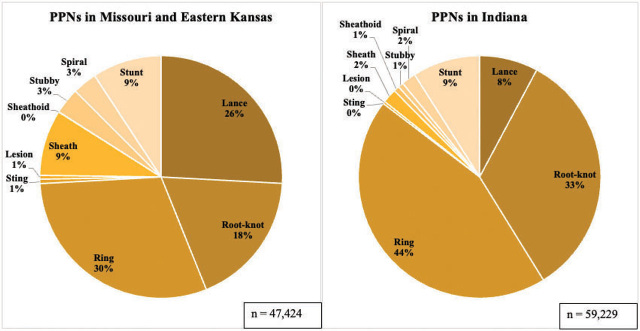
Distribution of plant-parasitic nematode species sampled from creeping bentgrass putting greens in Missouri and eastern Kansas in 2021 and Indiana in 2022 in two independent pie charts. Samples were collected during the months of April, June, August and October of 2021 and 2022, respectively. “n” indicates total PPNs represented within each chart.

*Overall PPN populations:* PPNs were more abundant in Indiana in 2022 than in MO/KC in 2021 (59,229 and 47,242, respectively) (Fig. 1). The lack of nematicide application in Indiana compared to MO/KC may be the cause of this disparity in populations (Table 1). Higher lance nematode populations were found in MO/KS than in Indiana (average of 1,225 and 462, respectively), potentially due to targeting sites with historically high lance nematode populations. Root-knot nematodes were more abundant in Indiana than in MO/KC (average of 1,997 and 857, respectively). Ring nematodes were more abundant in Indiana than in MO/KC (average of 2,617 and 1,432, respectively).

*Lance nematode population dynamics:* A significant depth-by-month interaction was observed in MO/KS lance populations in 2021 (*P* = 0.0487, Tables 4,5). Populations sampled from the upper 0–5 cm of the soil in October of 2021 in Missouri were significantly higher than any other depth and month (Tables 4,5). Lance populations sampled across Indiana in 2022 showed no significant depth-by-month interaction. Lance nematode populations were higher in August 2022 in Indiana than the other three months sampled (*P* = 0.003, Tables 4,6). As opposed to MO/KS, Indiana lance populations were statistically higher at the 5.1–10 cm sampling depth (*P* < 0.0001, Tables 4,6)

**Table 4:** Type III Tests of Fixed Effects for both Missouri and eastern Kansas in 2021 and Indiana in 2022. Data were analyzed using PROC GLIMMIX in SAS 9.4

	**Effect**	**Missouri**	**Indiana**
Lance	Depth	<.0001	<.0001
	Month	.0028	.0003
	Depth x Month	.0487	.4981
Root-Knot	Depth	.0004	<.0001
	Month	.7918	<.0001
	Depth x Month	.9998	.5897
Ring	Depth	<.0001	<.0001
	Month	<.0001	<.0001
	Depth x Month	.0001	<.0001
Free-Living	Depth	<.0001	<.0001
	Month	.0538	.0025
	Depth x Month	.0254	<.0001

**Table 5:** Missouri and eastern Kansas 2021 total nematode population densities by sampling depth and month with soil samples aggregated (100 cm^3^). Significance letters indicate significant differences between sampling depths by month analyzed within that individual species.

**Sampling Depth (cm)**											

**Species**	**Sampling Month**	**0–5**		**5.1–10**		**10.1–15**		**15.1–20**		**20.1–25**	
Lance	April	81.9^x^	bcde^y^	19.5	de	9.0	e	1.5	e	1.5	e
	June	144.0	b	85.5	bcde	18.0	e	10.5	e	10.5	e
	August	147.6	b	110.7	bc	53.1	cde	33.6	cde	21.3	cde
	October	309.0	a	105.6	bcd	27.0	cde	24.9	cde	10.8	e
Root-Knot	April	162.9	^*^	66.0	^*^	14.4	^*^	12.0	^*^	3.0	^*^
	June	97.5	^*^	39.0	^*^	4.5	^*^	3.0	^*^	13.5	^*^
	August	114.6	^*^	25.5	^*^	5.1	^*^	4.5	^*^	9.0	^*^
	October	185.4	^*^	62.1	^*^	11.4	^*^	8.1	^*^	15.9	^*^
Ring	April	21.9	c	27.0	c	21.0	c	3.0	c	3.9	c
	June	90.0	cb	64.5	cb	34.5	cb	13.5	c	18.0	c
	August	171.3	b	97.5	cb	37.2	cb	17.7	c	23.1	cb
	October	534.0	a	139.5	cb	62.7	cb	33.6	cb	18.3	cb
Free-Living	April	1188.9	c	238.5	c	80.4	c	43.8	c	43.8	c
	June	3178.5	b	429.0	c	189.0	c	114.0	c	90.0	c
	August	3187.2	b	306.7	c	154.2	c	108.3	c	143.1	c
	October	4653.0	a	351.8	c	186.9	c	100.5	c	119.8	c

x Mean of nematode population densities per 100 cm^3^.

y Means in the same column followed by the same letter are not different according to Fisher's protected LSD (*P* ≤ 0.05).

* Indicates no significant depth by month interaction.

**Table 6:** Indiana 2022 total nematode population densities organized by sampling depth and month with soil samples aggregated (100 cm^3^). Significance letters indicate significant differences between sampling depths by month analyzed within that individual species.

**Sampling Depth (cm)**											

**Species**	**Sampling Month**	**0–5**		**5.1–10**		**10.1–15**		**15.1–20**		**20.1–25**	
Lance	April	30.0^x^	^*^^y^	37.5	^*^	16.5	^*^	18.0	^*^	4.5	^*^
	June	16.5	^*^	63.0	^*^	13.5	^*^	4.5	^*^	3.0	^*^
	August	51.0	^*^	54.0	^*^	43.5	^*^	34.5	^*^	15.0	^*^
	October	10.5	^*^	19.5	^*^	16.5	^*^	6.0	^*^	4.5	^*^
Root-Knot	April	424.5	^*^	306.0	^*^	229.5	^*^	79.5	^*^	48.0	^*^
	June	145.5	^*^	67.5	^*^	19.5	^*^	4.5	^*^	0	^*^
	August	282.0	^*^	132.0	^*^	28.5	^*^	27.0	^*^	22.5	^*^
	October	70.5	^*^	55.5	^*^	18.0	^*^	9	^*^	7.5	^*^
Ring	April	189.0	bcd	58.5	cde	21.0	de	19.5	de	16.5	e
	June	337.5	b	153.0	cde	57.0	de	30.0	de	19.5	de
	August	1017.9	a	220.5	bc	121.5	cde	61.5	cde	33.0	de
	October	156.0	cde	57.0	cde	33.0	de	13.5	e	1.5	e
Free-Living	April	1591.5	b	316.5	c	129.0	c	73.5	c	58.5	c
	June	4144.5	a	405.0	c	132.0	c	60.0	c	28.5	c
	August	3559.5	a	343.5	c	160.5	c	61.5	c	60.0	c
	October	913.5	bc	147.0	c	67.5	c	43.5	c	30.5	c

x Mean of nematode population densities per 100 cm^3^.

y Means in the same column followed by the same letter are not different according to Fisher's protected LSD (P ≤ 0.05).

* Indicates no significant depth by month interaction.

**Figure 2:**
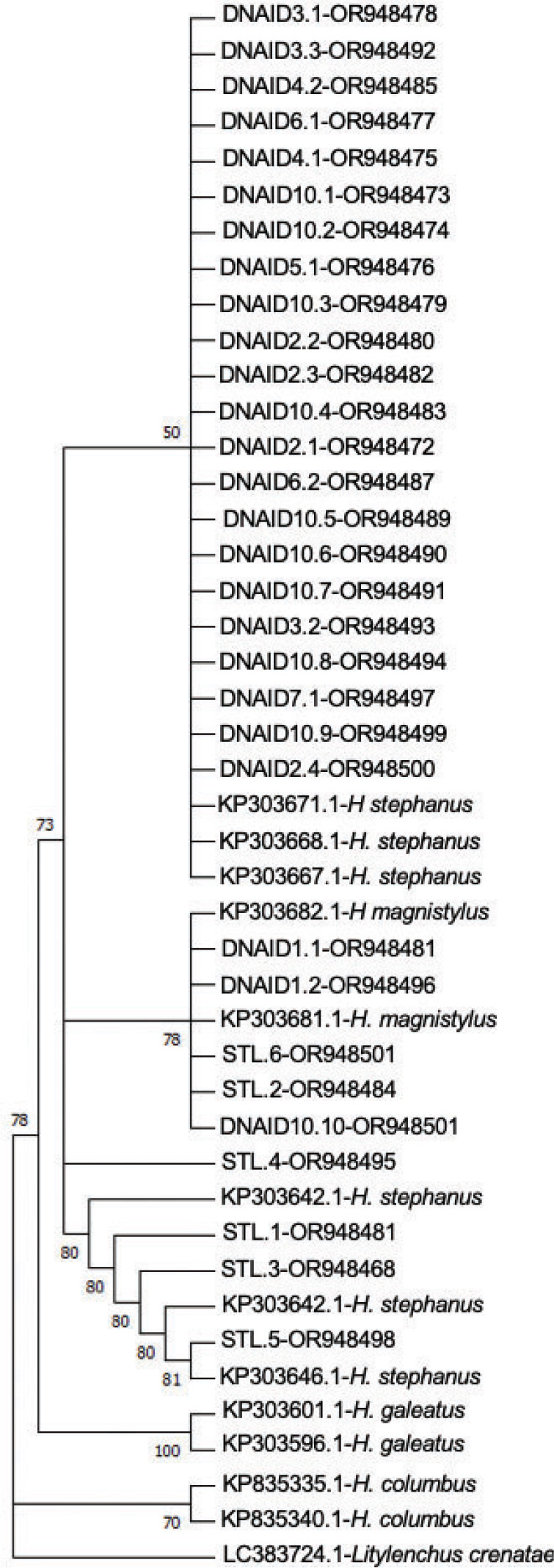
Phylogeny of the rDNA ITS region of *Hoplolaimus* spp. isolated from golf putting greens. Phylogenetic trees were constructed with the neighbor-joining algorithm using the Kimura two-parameter model with *Litylenchus* spp. (LC383724) as the outgroup. Bootstrap values are based on 1000 resamplings of the data set. DNAID codes correlate to Table 2.

**Figure 3:**
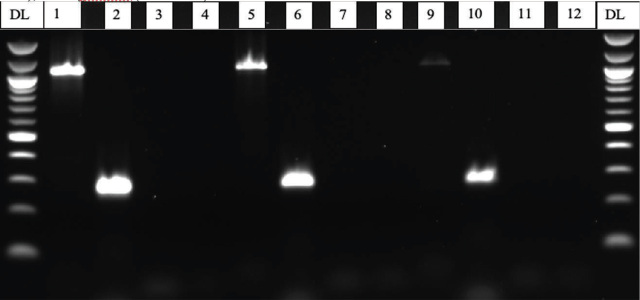
PCR results using *Hoplolaimus*-specific and *H. stephanus*, *H. columbus* and *H. galeatus*-specific primers. DL: DNA Ladder; 1: *Hoplolaimus* spp. (DNA ID:10); 2: *H. stephanus* (DNA ID:10); 3: *H. columbus* (DNA ID:10); 4 *H. galeatus* (DNA ID:10); 5: *Hoplolaimus* spp. (DNA ID:3); 6*: H. stephanus* (DNA ID:3); 7: *H. columbus* (DNA ID:3); 8 *H. galeatus* (DNA ID:3); 9: *Hoplolaimus* spp. (DNA ID:4); 10: *H. stephanus* (DNA ID:4); 11: *H. columbus* (DNA ID:4); and 12 *H. galeatus* (DNA ID:4).

*Hoplolaimus speciation and phylogeny:* In total, 31 ITS sequences were obtained using the DNA isolation and PCR amplification methods described above. Of the 31 sequences, 26 grouped in a clade with Genbank accessions of *H. stephanus,* and five grouped with *H. magnistylus* (Fig. 2). Amplicons from the HS-1f/HS-1r primer set designed by Ma et al. (19) amplified *H. stephanus* and no other species (Fig. 3). In reactions containing species-specific primer sets designed for *H. columbus* and *H. galeatus*, no amplicons were visualized. *H. magnistylus* was identified following successful amplification utilizing the genus-level Hoc-1f/LSUD-03r pairing and sequencing, but species-specific primers targeting *H. magnistylus* were not used for confirmation. DNA extracts of ITS sequences in the *H. magnistylus* clade did not have visualized amplicons after PCR with species-specific primers designed to amplify *H. galeatus*, *H. stephanus* or *H. columbus*.

**Figure 4:**
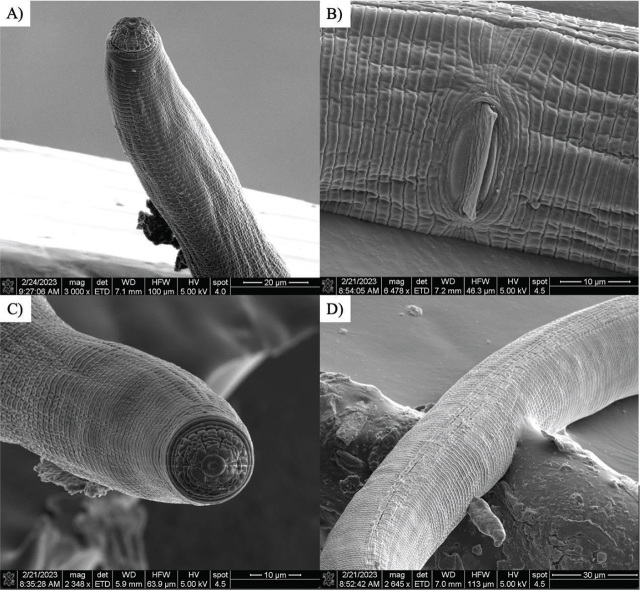
Scanning-electron micrographs of a lance nematode specimen collected form Site 5. A) four lip annules; B) the presence of an epiptygma; C) 25 longitudinal striae on the basal lip annule; and D) four lateral incisures.

Morphological traits characteristic of *H. stephanus* were observed with SEM. Specifically, the presence of four lip annules (Fig. 4A), the presence of an epiptygma (Fig. 4B), 25 longitudinal striae on the basal lip annule that were within the range of that of *H. stephanus* and not *H. galeatus* (Fig. 4C) and four lateral incisures (Fig. 4D) (16,32).

*Root-knot nematode population dynamics:* Populations of root-knot nematodes in MO/KC were not significantly different between sampling months (Tables 4,5). Populations were higher at the 0–5 cm sampling depth in 2021 than all other depths, and the 5.1–10 cm depth had higher populations than deeper samples (*P* = 0.0004, Table 5). In contrast, root-knot nematode population levels in Indiana were significantly higher in April 2022 than any other month in 2022 (*P* < 0.0001, Tables 4,6). Root-knot nematode populations in Indiana were higher at the 0–5 cm and 5.1–10 cm sampling depths than at the lowest three depths (*P* < 0.0001, Tables 4,6).

**Figure 5:**
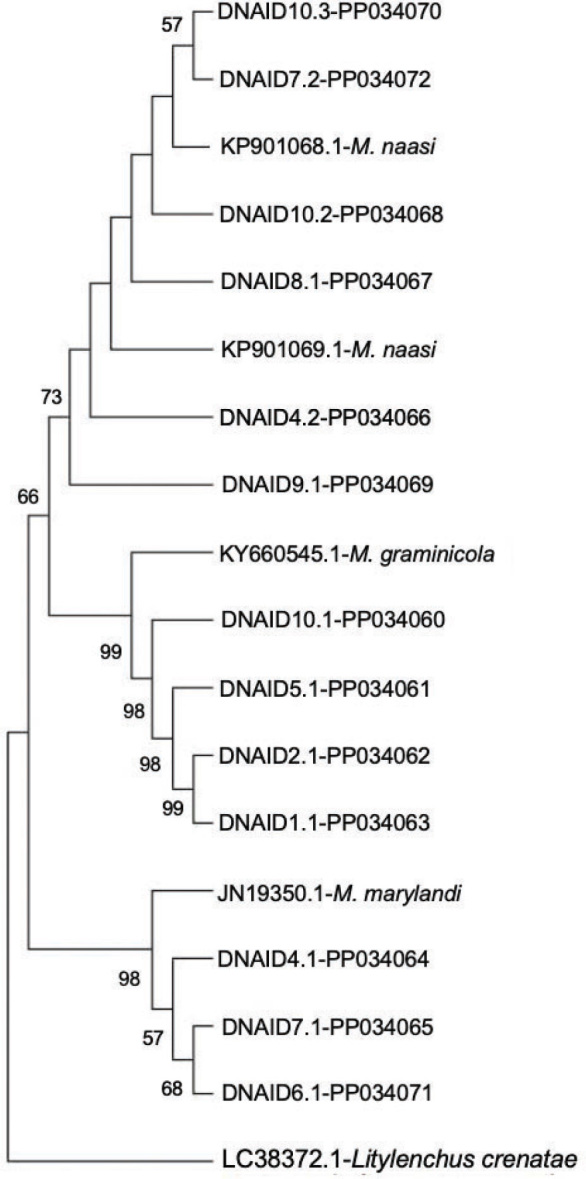
Phylogeny of molecularly characterized *Meloidogyne* spp. isolated from golf coursed based on D2/D3 28S genes. phylogenetic trees were constructed with the neighbor-joining algorithm using the Kimura two-parameter model with *Litylenchus* spp. (LC383724) as the outgroup. Bootstrap values are based on 1000 resamplings of the data set and displayed near branch nodes. DNAID codes correlate to Table 2.

**Figure 6:**
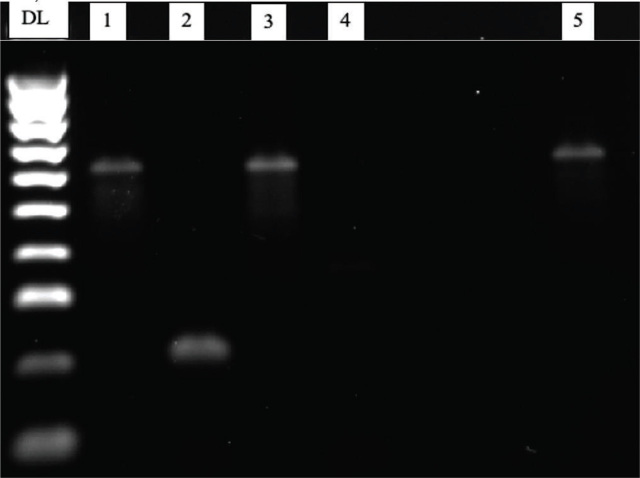
PCR results using *Meloidogyne*-specific and *M. naasi* and *M. marylandi*-specific primers. DL: DNA Ladder; 1: *Meloidogyne spp. (DNA ID:9); 2: M. naasi* (DNA ID:9); 3: *Meloidogyne* spp. (DNA ID:4); 4: *M. marylandi* (DNA ID:4); and 5: *Meloidogyne* spp.(DNA ID:4).

*Meloidogyne speciation and phylogeny:* In total, 13 28S D2/D3 sequences were obtained using the primer pair RK28SF/MR as described by Ye et al. (36). Of the 13 D2/D3 sequences obtained, six grouped in a clade with Genbank accessions of *M. naasi*, four with *M. graminicola* and three with *M. marylandi* (Fig. 5). Additional species confirmation with species-specific primers was provided for *M. naasi* and *M. marylandi*. (Fig. 6). *M. graminicola* was not tested with species-specific primer sets for gel confirmation in this study.

*Other nematode population trends:* Free-living, or non-plant parasitic nematodes, were the most abundant nematode in these samples at 10 times or more the number of PPNs found. The most abundant PPN in MO/KC and Indiana was the ring nematode (*Criconemoides* spp.) (Fig. 1). In both MO/KC and Indiana, free-living and ring nematodes had a statistically significant depth-by-month interaction (*P* = 0.0254, *P* = 0.0001; *P* < 0.0001, *P* < 0.0001, respectively, Tables 4–6). In MO/KC, ring and free-living nematodes were significantly more abundant in the 0–5 cm range during October than any other depth–month combination (Tables 4,5). In Indiana, higher ring nematode populations were observed during August at a depth of 0–5 cm than any other depth–month combination (Tables 4,6). In Indiana, free-living nematodes were most abundant during the months of June and August at the 0–5 cm sampling depth, with no significant difference between the two (Tables 4,6).

## Discussion

Populations of lance, root-knot, ring and free-living nematodes fluctuated throughout the season across all sampling sites and sampling depths. Other researchers have also demonstrated considerable variation in plant-parasitic nematode (PPN) populations seasonal dynamics (2,10,18,22,25,26). Some trends agree with one another despite differences in their cropping systems, and some are vastly different. In turfgrass, lance nematode population dynamics have been tied to soil temperature and natural reproductive cycles, while some studies demonstrated no association (18,26,33). The relationship between the decline of root-biomass and lance populations indicates a self-regulating feedback loop of PPN population density. As the competition for food increases through changes in nutrient density and root-decline, a constraint on the reproductive factor of the PPNs is more prevalent (10). Evidence also indicates a potential shift to endoparasitism when soil temperatures reach a certain threshold (26), which was not accounted for in this study. Lance nematode populations steadily rose throughout the year, and a reduction in lance nematodes in midsummer following root decline was not observed in this study. Lance nematode populations in Indiana in 2022 peaked significantly in August, when soil temperatures most likely are not conducive for creeping bentgrass root development and subsequent increases in lance nematode populations.

On average, root-knot nematode populations were below the threshold in which turfgrass managers are recommended to apply a nematicide (37). Significantly more root-knot populations were found in MO/KC at the 0–5 cm depth, and it is widely reported that these species tend to aggregate at this depth. The presence of significantly more root-knot nematodes in April agrees with an early-year population density described by Barker (2) but contrasts with populations recorded by Sasser (25). In his study, Barker indicates moisture levels were high enough in January to induce egg hatch in root-knot populations in February, so populations recorded then would be higher, followed by a reduction as competition for food increases. Sasser observed population peaks in November, but indicated this population fluctuation may be specific to the host crop. Additionally, Sasser sampled only during the months of February, May and November of one year, and March of the following year. The time gaps between these selected sampling months could be a time in which *Meloidogyne* spp. populations peaked.

Populations of ring nematodes shared a depth-by-month interaction, but the month in which the most ring nematodes were found in the 0–5 cm range differed between MO/KC and Indiana. While ring populations peaked in August and October in Indiana and MO/KC, respectively, Davis (6) reported ring nematode populations on a mixed bentgrass/bluegrass green in Chicago peaked in June. Davis was able to analyze populations of ring nematodes at the same sampling site through two years and found their populations did not peak in the same month two years in a row. However, Davis noted that trends did not agree year over year, and populations analyzed during the same year on two different greens within the same golf course changed together. Wick (33) observed populations on the same green did not change synchronously, and thus discredits evidence that population dynamics may be extrapolated across greens on the same course.

The disagreement of this study with other similar research indicates attempting to predict PPN populations based solely upon time of the year may be ineffective. In addition, Settle (26) describes the spatial aggregation of PPN communities across a putting green may vastly change the populations observed. Because of this, efforts to predict overall PPN populations within a green may be ineffective with current sampling methods, as one area of the putting green may contain significantly more PPNs than an area just a few meters away. The results of this study indicate a spring nematicide application would lower the PPN's ability to increase their populations throughout the summer, dependent on the nematicides ability to remain undegraded and maintain residual efficacy. However, differences in the half-life of common nematicides, e.g., the relatively short half-life of abamectin compared to longer half-life of fluopyram, is an important consideration when deciding which chemistry to employ (23,35). The presence of significantly more lance and ring nematodes in MO/KC found at 0–5 cm during October in this study indicates an additional early fall nematicide application date may be necessary to suppress these population peaks and potentially reduce these populations the following year. If turfgrass damage in Indiana can be accurately correlated to high lance nematode populations, a late season nematicide application may be more effective in controlling lance populations the following year. However, to target and control other PPNs throughout the summer season, an early spring application may be more effective.

*Hoplolaimus* spp. and *Meloidogyne* spp. exhibited differences between species across and within sampling sites. *Meloidogyne* spp. analyzed in this study were more diverse than *Hoplolaimus* spp. *M. naasi*, *M. marylandi* and *M. graminicola* are known parasites of creeping bentgrass that were found in this study (21,36). The geographic distribution and host species of several *Meloidogyne* species seemed to overlap, indicating root-knot species have a mixed distribution and may commonly form a species complex on golf greens. Ye et al. (36) described the most prominent species on a mix of warm and cool season greens in North Carolina were *M. marylandi* and *M. graminis*, whereas McClure et al. (21) described the most prominent species obtained from golf course greens in the Western United States as *M. naasi*. *M. naasi* is a well-described parasite of turfgrass in the United States, and McClure et al. (21) described that two different *Meloidogyne* species (*M. graminis* and *M. marylandi*) cohabitated a single green in California, suggesting species complexes of *Meloidogyne* on golf greens are common.

With ITS sequence data as sole evidence of speciation, this is the first report of *H. magnistylus* associated with bentgrass putting greens to the authors' knowledge. The species was putatively found in a mixed population with *H. stephanus* at a site in both Missouri and Indiana and alone at another site in Indiana. The species has previously been reported in Arkansas, Illinois and Tennessee parasitizing corn, cotton, soybean and willow (5,16). Morphologically, it differs from *H. concaudajuvencus* by the possession of rounded tails in second-stage juveniles vs. conically pointed tails with acute termini, having fewer subdivisions in female basal lip annules, and the greater distance from female anterior end to posterior end of esophageal lobes (24). Further micromorphological observation is necessary to confirm this result.

*H. stephanus* was the most numerous lance nematode characterized on bentgrass putting greens in the Midwest. Ma et al. (19) described an under-representation of *H. stephanus* in the literature, indicating reports of *H. galeatus* as the most often reported species responsible for damage on golf putting greens may be inaccurate. The over-representation of *H. galeatus* may be due to an overlap of host plants and geographic distribution and the difficulty of using morphological traits for speciation. Holguin et al. (16) noted that while their host plant specificity may overlap, geographic distribution may be the driving force for species presence. Specifically, while *H. stephanus* and *H. galeatus* were both found to be parasites of *Agrostis* spp., *H. stephanus* was the only lance nematode found on bentgrass greens in Ohio and Kansas, and *H. galeatus* was the only lance nematode found in South Carolina and Florida in their study. In South Carolina and Florida, *H. galeatus* was found parasitizing warm-season turfgrass species and cool-season *Agrostis* spp. Recently, *H. stephanus* was discovered parasitizing creeping bentgrass in Georgia using similar molecular and morphological identification diagnostic techniques to what was used in this study (17). Further characterization of lance nematodes collected from golf courses across the United States is needed to assess whether lance nematode species distributions are determined by geography or by host range.

This study reiterates literature on *Hoplolaimus stephanus*, regarding it as an under-represented, yet seemingly widely distributed, lance nematode species within the United States (16,19,27,32). The presence of multiple lance and root-knot species, some obtained from the same putting green, indicates a potential overlap in both geographic distribution and host preference. The broad geographic distribution could be due to several factors described by McClure et al. (21) such as topdressing with nematode contaminated sand from other regions, or golf shoes and/or clubs introducing juveniles or eggs when playing golf. Additionally, putting green proximity to agricultural systems and/or native plant species where particular plant-parasitic nematode species may be abundant and exhibit a wide host range could further expose the putting green to the introduction of detrimental, native nematodes over time.
